# Emphysema Distribution and Diffusion Capacity Predict Emphysema Progression in Human Immunodeficiency Virus Infection

**DOI:** 10.1371/journal.pone.0167247

**Published:** 2016-11-30

**Authors:** Janice M Leung, Andrea Malagoli, Antonella Santoro, Giulia Besutti, Guido Ligabue, Riccardo Scaglioni, Darlene Dai, Cameron Hague, Jonathon Leipsic, Don D. Sin, SF Paul Man, Giovanni Guaraldi

**Affiliations:** 1 Centre for Heart Lung Innovation, University of British Columbia, Vancouver, BC; 2 Division of Respiratory Medicine, Department of Medicine, St. Paul’s Hospital, Vancouver, BC; 3 Modena HIV Metabolic Clinic, University of Modena and Reggio Emilia, Modena, Italy; 4 PROOF Centre for Excellence, University of British Columbia, Vancouver, BC; 5 Department of Radiology, University of British Columbia, Vancouver, Canada; Lee Kong Chian School of Medicine, SINGAPORE

## Abstract

**Background:**

Chronic obstructive pulmonary disease (COPD) and emphysema are common amongst patients with human immunodeficiency virus (HIV). We sought to determine the clinical factors that are associated with emphysema progression in HIV.

**Methods:**

345 HIV-infected patients enrolled in an outpatient HIV metabolic clinic with ≥2 chest computed tomography scans made up the study cohort. Images were qualitatively scored for emphysema based on percentage involvement of the lung. Emphysema progression was defined as any increase in emphysema score over the study period. Univariate analyses of clinical, respiratory, and laboratory data, as well as multivariable logistic regression models, were performed to determine clinical features significantly associated with emphysema progression.

**Results:**

17.4% of the cohort were emphysema progressors. Emphysema progression was most strongly associated with having a low baseline diffusion capacity of carbon monoxide (DLCO) and having combination centrilobular and paraseptal emphysema distribution. In adjusted models, the odds ratio (OR) for emphysema progression for every 10% increase in DLCO percent predicted was 0.58 (95% confidence interval [CI] 0.41–0.81). The equivalent OR (95% CI) for centrilobular and paraseptal emphysema distribution was 10.60 (2.93–48.98). Together, these variables had an area under the curve (AUC) statistic of 0.85 for predicting emphysema progression. This was an improvement over the performance of spirometry (forced expiratory volume in 1 second to forced vital capacity ratio), which predicted emphysema progression with an AUC of only 0.65.

**Conclusion:**

Combined paraseptal and centrilobular emphysema distribution and low DLCO could identify HIV patients who may experience emphysema progression.

## Introduction

Chronic obstructive pulmonary disease (COPD) is characterized by small airway remodeling and emphysematous lung destruction and is an important comorbidity in patients living with human immunodeficiency virus (HIV)[1, 2] contributing to substantial respiratory symptom burdens[3, 4]. While spirometry is currently the accepted method for diagnosing COPD[5], evidence from HIV-specific studies suggests that it may be inadequate in explaining the degree of respiratory impairment observed in this population. For instance, St. George’s Respiratory Questionnaire (SGRQ) scores, which measure respiratory-related health status[6], can demonstrate significant impairment in HIV-infected subjects with otherwise normal spirometry[4]. Similarly, HIV patients with severe emphysema burdens as visualized on computed tomographic (CT) scanning can also have surprisingly well-preserved spirometry[7]. This stands in contrast to HIV-uninfected subjects in whom emphysema quantitation correlates strongly with forced expiratory volume in 1 second (FEV1) measurements[8]. Why spirometry fails to capture the scope and severity of obstructive lung disease specifically in HIV has yet to be answered.

The search for alternate measurements of COPD severity in HIV has led to investigations of radiographic emphysema. In large, general population COPD cohorts, the presence of emphysema on CT imaging has been associated with increased dyspnea scores and reduced 6 minute walk distances[9], as well as with worse SGRQ scores[10], suggesting that emphysema may be a clinically meaningful marker of disease severity. In HIV-specific populations, the investigation of emphysema has been limited to cross-sectional, descriptive studies that have reported a range of emphysema prevalence from 26% to 53%[7, 11–14]. Longitudinal studies looking at the progression of emphysema over time and the risk factors associated with rapid emphysema progression in HIV have yet to be performed. In this study, we used a large, HIV-infected population with serial chest CT images to determine a predictive model of emphysema progression.

## Methods

### HIV Study Population

The study cohort was drawn from an unselected outpatient HIV metabolic clinic at the University of Modena and Reggio Emilia, Modena, Italy[7]. Inclusion criteria to the cohort were: serologically documented HIV-1 infection, age >18 years, and >18 months of antiretroviral (ART) exposure. Respiratory symptoms were not a requirement to enter the study cohort. Demographic and clinical data were collected on the same date as the CT scans, including age, sex, current smoking status, amount of smoking, ART use, body mass index (BMI), and comorbidities. Pregnant women were excluded from the cohort. Written informed consent was obtained from all participants. The institutional ethics review committee at the University of Modena and Reggio Emilia (ComitatoEticoProvinciale) approved the study.

### Chest CT Imaging

CT scans were performed as part of the study protocol and not for clinical reasons. Scans were performed using a 64-slice scanner (GE Medical Systems, Milwaukee, Wisconsin). Images were obtained during a single breath hold using 320 mAs and 140kV. A section thickness of 2.5 mm, a field of view of 40 cm, and a matrix of 512x512 were used to reconstruct the raw image data, yielding a nominal pixel size of 0.39 mm^2^ and a voxel of 0.4 mm^3^. Repeat chest CTs were performed at 1–2 year intervals as per the study protocol. Patients with ≥2 chest CTs were included in the final analysis.

### Qualitative Emphysema Scoring

**Fig 1:**
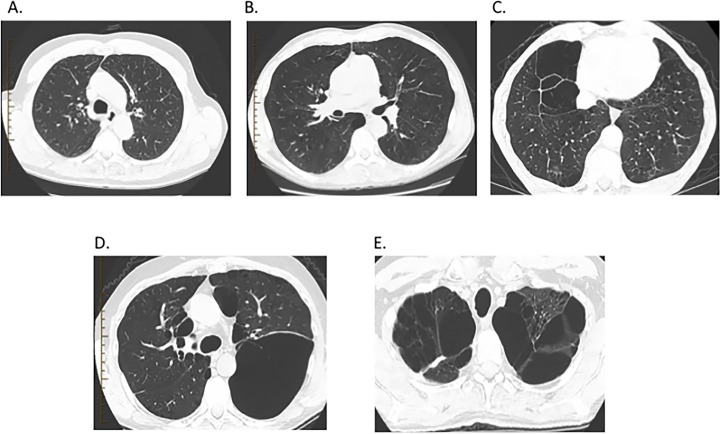
Representative images of emphysema scoring are shown. A) 0 = no emphysema. B) 1 = 1–25% emphysema. C) 2 = 26–50% emphysema. D) 3 = 51–75% emphysema. E) 4 = 76–100% emphysema.

**Fig 2:**
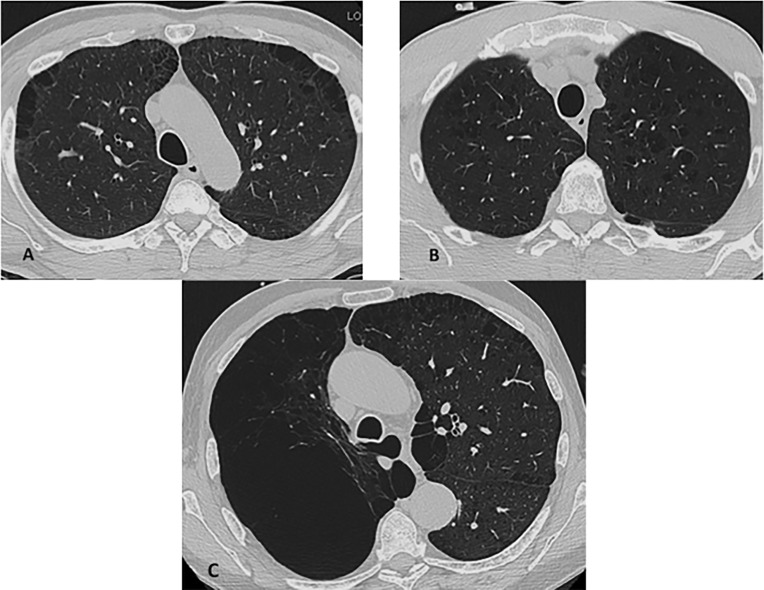
Representative images of emphysema distribution are shown. A) Paraseptal distribution. B) Centrilobular distribution. C) Combination paraseptal and centrilobular distribution.

Three radiologists (GB, GL, and RS with 5, 20, and 5 years experience, respectively) blinded to clinical data reviewed images using an offline CT workstation (AW4.4, GE Healthcare, Milwaukee, WI). Qualitative emphysema scoring was performed according to a modified method of Kazerooni*et al*.[15] which has been used to score similar images obtained in the COPDGene cohort[16]. In this method, a score was assigned to the 5 lobes and the lingula based on the percentage of emphysema visualized: 0 –no emphysema; 1–1–25% emphysema; 2–26–50% emphysema; 3–51–75% emphysema; 4–76–100% (Fig 1). A total emphysema score was then created by summing the scores of the 5 lobes and the lingula. An individual was designated as having emphysema progression if their total emphysema score increased over the trial period over the baseline total emphysema score. Images were also analyzed to determine the distribution of emphysema, whether paraseptal, centrilobular, or a combination of paraseptal and centrilobular (Fig 2). Of note, images were also assessed for pulmonary fibrosis; however, only three patients were found to have reticular abnormalities and no patients were found to have honeycombing.

### Pulmonary Function Testing

Pulmonary function testing (spirometry, diffusion capacity of carbon monoxide [DLCO], and lung volumes) was performed on the same day as the first chest CT scan using a Jaeger^®^FlowScreen™ spirometer and a Jaeger^®^MasterScreen™ plethysmograph according to recommendations outlined by the American Thoracic Society and European Respiratory Society[17]. Inhaled salbutamol (400 ug) was provided to subjects demonstrating FEV1/forced vital capacity (FVC) ratios <70%. Values for FEV1%Predicted, FVC %Predicted, and FEV1/FVC reported in the study are therefore post-bronchodilator values. DLCO values were not corrected for hemoglobin or carboxyhemoglobin, or alveolar volume.

### Laboratory Studies

After an overnight fast, blood was collected using standard venipuncture methods on the same day as the chest CT scan. C-reactive protein (CRP), total protein, albumin, cholesterol, bilirubin, liver enzymes, glomerular filtration rate, CD4 and CD8 cell count, and quantitative plasma HIV-1 RNA levels were measured. These measurements were performed at the metabolic clinic of the University of Modena and Reggio Emilia.

### Statistical Analysis

Emphysema progression was considered the outcome variable of interest. Univariate analysis using a Wilcoxon rank-sum test for continuous variables or a Fisher’s exact test for categorical variables was initially performed to determine baseline characteristics (those obtained on the date of the first chest CT scan available) associated with future emphysema progression. All variables with a p-value <0.20 were then included in a multivariable logistic regression analysis to determine the significant factors associated with emphysema progression. Variables were removed from the logistic regression model using a backward stepwise regression until the lowest Akaike information criterion (AIC) was obtained. Both unadjusted and adjusted (based on the final multivariable model) odds ratios (OR) with 95% confidence intervals (CI) for emphysema progression were calculated for the variables. The final multivariable model was then used to generate a receiver operating characteristic (ROC) curve for the prediction of emphysema progression with area under the curve (AUC) statistics calculated. Significance was determined as a p-value <0.05. All statistical analyses were performed using R (version 3.2.3).

## Results

### Baseline Demographic Characteristics of the HIV Cohort

Table 1 outlines the baseline demographic characteristics of the 345 enrolled patients. The cohort was predominantly male (90%) and all participants were on ART. The median current CD4 cell count was relatively preserved at 577 cells/mm^3^ and 77% had undetectable HIV viral loads (considered as <40 copies/mL). Just under 80% of the cohort were either current or former smokers with a median smoking history of 17 pack-years. The range of the time interval between first and last CT scans was from 1 to 7 years (median 3.93 years, interquartile range 2.17 to 5.39 years).

**Table 1:** Baseline Demographic Characteristics of the Study Cohort (n = 345).

Variable	Median (Interquartile Range) or n (%)
Age (years)	49 (45, 53)
Male Sex (%)	311 (90%)
BMI (kg/m^2^)	23.8 (21.9, 26.0)
Current CD4 Count (cells/mm^3^)	577 (430, 746)
Nadir CD4 Count (cells/mm^3^)	188 (65, 290)
Viral Load <40 copies/mL	267 (77%)
Smoking Status	
• Never • Former • Current, <10 cigarettes/day • Current, ≥10 cigarettes/day • Not available	• 64 (19%) • 101 (29%) • 70 (20%) • 96 (28%) • 14 (4%)
Smoking Pack-Years	17 (5, 28)
Ever Injection Drug Use (%)	88 (25.5%)
Men Having Sex With Men (MSM) (%)	139 (40.3%)
FEV1/FVC (%)	79.3 (75.6, 83.0)
FEV1%Predicted	106.1 (96.7, 116.8)
FVC %Predicted	109.0 (98.3, 118.0)
TLC %Predicted	114.6 (106.1, 122.9)
DLCO %Predicted	75.2 (65.7, 87.2)
Presence of Emphysema (%)	143 (41%)
CT Emphysema Score	0 (0, 3)

Abbreviations: BMI–body mass index; FEV1 –forced expiratory volume in 1 second; FVC: forced vital capacity; TLC–total lung capacity; DLCO–diffusion capacity of carbon monoxide; CT–computed tomography

### Characteristics Associated with Emphysema Progression in HIV

Sixty (17.4%) were determined to be emphysema progressors and 285 (82.6%) were determined to be emphysema non-progressors. Of note, the median time interval between first and last CT scans was not different between progressors and non-progressors (3.63 vs. 3.62 years, p = 0.965). All characteristics distinguishing progressors from non-progressors with a p-value <0.20 in a univariate analysis are displayed in Table 2, while characteristics with p-values ≥0.20 are displayed in S1 Table. Specific ART drug use by emphysema progression group is shown in S2 Table; there were no significant differences between the two groups for each ART drug. Subjects with a higher burden of emphysema on baseline CT scans were more likely to be progressors (p<0.001), as were subjects who demonstrated a distribution of emphysema that was both centrilobular and paraseptal (p<0.001). Current smokers who were smoking ≥10 cigarettes/day (p<0.001) and those who had a higher smoking pack-year history (p<0.001) were also more likely to be progressors. More emphysema progressors had a history of injection drug use, while more emphysema non-progressors were men having sex with men (MSM) (p = 0.043). Lung function parameters such as DLCO %Predicted (p<0.001) and FEV1/FVC (p<0.001) were also significantly associated with emphysema progression. Emphysema progression was notably not associated with current CD4 cell count, nadir CD4 cell count, current CD4:CD8 ratio, HIV viral load, ART classes, or duration of ART exposure.

**Table 2:** Characteristics Differentiating Emphysema Progressors From Non-Progressors.^#^

Variable	Progressor(n = 60)	Non-Progressor (n = 285)	p-value*
Emphysema Score	4 (1, 6)	0 (0, 2)	<0.001
Emphysema Distribution			<0.001
• Centrilobular • Paraseptal • Centrilobular and Paraseptal	• 3 (5%) • 9 (15%) • 37 (62%)	• 40 (14%) • 34 (12%) • 41 (14%)	
Smoking Status			<0.001
• Never or former • Current, <10 cigarettes/day • Current, ≥10 cigarettes/day • Not available	• 15 (25%) • 11 (18%) • 32 (53%) • 2(3%)	• 150 (53%) • 59 (21%) • 64 (22%) • 12 (4%)	
Smoking Pack-Years	22.5 (15.3, 31.8)	15.0 (1.0, 26.0)	<0.001
Baseline DLCO %Predicted	64.8 (54.3, 78.1)	77.5 (68.2, 88.0)	<0.001
Baseline FEV1/FVC (%)	77.05 (72.4, 80.4)	79.8 (76.4, 83.6)	<0.001
BMI (kg/m^2^)	22.9 (20.7, 24.6)	24.0 (22.0, 26.2)	0.011
Male Sex (%)	59 (98%)	252 (88%)	0.016
Albumin (g/dL)	4.6 (4.3, 4.8)	4.7 (4.5, 4.9)	0.038
HIV Risk Factor			0.043
• Injection Drug Use • MSM • Heterosexual • Other	• 21 (35.0%) • 15 (25.0%) • 17 (28.3%) • 7 (11.7%)	• 67 (23.5%) • 124 (43.5%) • 69 (24.2%) • 25 (8.8%)	
Baseline TLC %Predicted	117.2 (111.4, 128.0)	114.0 (105.9, 122.2)	0.067
Duration of HIV (Months)	206 (155, 268)	198 (129, 246)	0.195

Abbreviations: DLCO–diffusion capacity of carbon monoxide; FEV1 –forced expiratory volume in 1 second; FVC–forced vital capacity; BMI–body mass index; TLC–total lung capacity

^#^Values are recorded as either median (interquartile range) or n (%).

*P-values obtained either through Wilcoxon rank sum testing for continuous variables or Fisher’s exact test for categorical variables.

### Logistic Regression Model

Unadjusted OR for emphysema progression using the variables from Table 2 are provided in Table 3. These variables were then entered into a multivariable logistic regression model. Based on the lowest AIC, the following variables were removed stepwise to generate the final model: duration of HIV infection, TLC %Predicted, FEV1/FVC, smoking status, and emphysema score. The adjusted odds ratios based on the final model are also shown in Table 3. In this model, every 10% increase in DLCO %Predicted resulted in an OR of 0.58 for emphysema progression (95% confidence interval [CI] 0.41–0.81, p<0.001). Having a combination centrilobular and paraseptal distribution of emphysema resulted in an OR of 10.60 for emphysema progression (95% CI 2.93–48.98, p<0.001). Other variables that retained statistical significance in the multivariable model included male sex, smoking pack-years, and BMI.

**Table 3:** Unadjusted and Adjusted Odds Ratios for Emphysema Progression.

Variable	Unadjusted		Adjusted	
	OR (95% CI)	p-value	OR (95% CI)*	p-value
Male Sex	7.73 (1.61–138.77)	0.046	16.37 (1.70–510.05)	0.042
Emphysema Distribution				
• None (Reference)				
• Centrilobular • Paraseptal • Centrilobular and Paraseptal	• 1.16 (0.25–3.92) • 4.09 (1.54–10.65) • 13.95 (6.76–30.90)	• 0.827 • 0.004 • <0.001	• 1.04 (0.04–9.28) • 1.80(0.19–13.48) • 10.60 (2.93–48.98)	• 0.976 • 0.577 • <0.001
Smoking Status				
• Never (Reference)				
• Former • Current, <10 cigarettes/day • Current, ≥10 cigarettes/day	• 2.97 (0.91–13.32) • 3.56 (1.03–16.45) • 10.50 (3.53–45.26)	• 0.100 • 0.063 • <0.001	----	----
Smoking Pack Years (per 10 pack-years)	1.34 (1.13–1.59)	<0.001	1.58 (1.07–2.39)	0.024
DLCO %Predicted (per 10%)	0.65 (0.51–0.81)	<0.001	0.58 (0.41–0.81)	<0.001
BMI	0.89 (0.81–0.97)	0.014	0.82 (0.67–0.98)	0.036
Baseline FEV1/FVC (%) (per 10%)	0.48 (0.32–0.71)	<0.001	----	----
Emphysema Score	1.59 (1.41–1.81)	<0.001	----	----
Log Albumin (per 0.1 units)	0.76 (0.44–1.25)	0.252	0.51 (0.23–1.52)	0.130
HIV Risk Factor				
• Other (Reference)				
• Injection Drug Use • MSM • Heterosexual	• 1.12 (0.44–3.13) • 0.43 (0.16–1.23) • 0.88 (0.34–2.50)	• 0.820 • 0.098 • 0.800	• 0.40 (0.07–2.47) • 0.20 (0.02–1.66) • 6.01 (0.96–48.14)	• 0.316 • 0.069 • 0.140
TLC %Predicted (per 10%)	1.22 (0.97–1.54)	0.096	----	----
Duration of HIV (per 12 months)	1.03 (0.99–1.08)	0.164	----	----

Abbreviations: OR: odds ratio; CI: confidence interval; DLCO: diffusion capacity of carbon monoxide; BMI: body mass index; MSM: men having sex with men; TLC: total lung capacity

*Smoking status, FEV1/FVC, TLC %Predicted, duration of HIV (months), and emphysema score were removed to generate the final model by lowest AIC.

### ROC Curves

**Fig 3:**
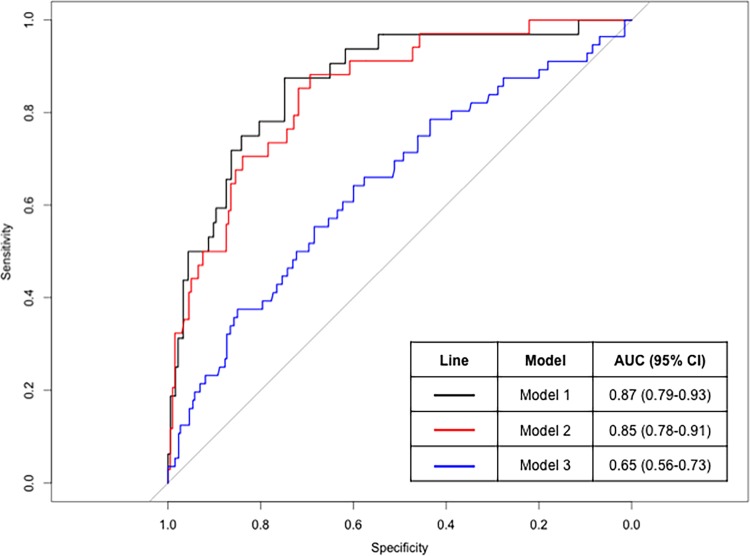
Receiver operating characteristic curves for emphysema progression. Model 1 contained variables in the multivariable logistic regression model that retained statistical significance (sex, emphysema distribution, smoking pack-years, DLCO %Predicted, and BMI). Model 2 contained only emphysema distribution and DLCO %Predicted, the two variables with the most statistical significance in the final multivariable model. Model 3 consisted only of FEV1/FVC.

ROC curves for emphysema progression are depicted in Fig 3 for three models. Model 1 contained variables in the multivariable logistic regression model that retained statistical significance (sex, emphysema distribution, smoking pack-years, DLCO %Predicted, and BMI). Model 2 contained only emphysema distribution and DLCO %Predicted, the two variables with the most statistical significance in the final multivariable model. To compare these models with commonly used spirometry measures, Model 3 consisted only of FEV1/FVC. Model 1 had an AUC statistic of 0.87 (95% confidence interval [CI] 0.79–0.93) to predict emphysema progression. Model 2 carried a nearly equivalent AUC statistic of 0.85 (95% CI 0.78–0.91). The AUCs of Model 1 and Model 2 were not statistically significantly different from each other (p = 0.697). These performances were a significant improvement over Model 3, which had an AUC statistic of 0.65 (95% CI 0.56–0.73). The AUCs of Models 1 and 2 were statistically significantly different from the AUC of Model 3 (p<0.001 for both Models).

## Discussion

We report here the first algorithm for the prediction of emphysema progression in an HIV-infected cohort. Using just two parameters that are relatively easy to obtain in a clinical setting (DLCO and emphysema distribution), we were able to predict emphysema progression over a seven-year period with an AUC statistic of 0.85. This was in contrast to the poor predictive abilities of spirometry, again demonstrating its limitations in assessing the severity or progression of COPD in HIV. Chest CT imaging and DLCO measurements may be useful adjunct tools for the HIV clinician to employ for risk stratification for lung disease. These tools may be particularly helpful in patients in whom spirometry appears incongruent with symptoms.

The importance of emphysema distribution in predicting emphysema progression was a novel finding. In non-HIV populations, certain patterns of emphysema distribution have been associated with worse clinical outcomes. In the COPDGene study, subjects with paraseptal distribution of emphysema had the most severe Modified Medical Research Council dyspnea scores, 6 minute walk distances, and number of COPD exacerbations compared to subjects with centrilobular or panlobular distribution[18]. On the other hand, another recent study demonstrated that only centrilobular and panlobular distribution were associated with increased dyspnea and shorter 6 minute walk distances[19]. Few studies have assessed subjects with co-existing patterns of distribution; therefore, it is difficult to determine whether a combination of centrilobular and paraseptal distribution such as found in our study has the greatest clinical impact. While centrilobular emphysema is often associated with cigarette smoke and dust inhalation[20], paraseptal emphysema may be the result of reduced blood flow in the periphery of the lung[21]. We speculate that the particular pathology of emphysema in HIV that appears in such accelerated form may be the synergistic product of these two processes, toxic particle exposure in the respiratory bronchioles and vascular insufficiency in the distal acinus. Notably, 22.6% of the HIV cohort had a combination centrilobular and paraseptal distribution. Having a combination of both distributions was also associated with severity of disease, therefore distribution may be a marker for patients with an accelerated phenotype of COPD.

What remains unanswered by our study is the mechanism by which emphysema progresses in HIV. A number of theories have been proposed as to why COPD occurs in accelerated or accentuated fashion in HIV, such as chronic inflammation and immunosuppression[14, 22], long term exposure to ART[23], and respiratory infections including *Pneumocystis jirovecii* pneumonia (PJP)[24, 25]. Interestingly, none of these theories were supported by our study. Inflammatory markers such as CRP were not elevated in emphysema progressors, nor were emphysema progressors more likely to have lower CD4 cell counts, lower CD4:CD8 ratios, or detectable HIV viral loads. Similarly, there was no relationship between emphysema progression and ART exposure and there were too few events of PJP and other pneumonia in our cohort (all of whom were on ART with many having adequate CD4 cell counts) to report a meaningful statistical relationship.

While it is clear that the subset of HIV patients who develop emphysema appear to have an ongoing trigger promoting the progression of disease, what exactly this trigger is has yet to be defined. Recent theories proposing an accelerated cellular aging process in these individuals are thought-provoking[26, 27] and work linking aging with COPD and emphysema may yet reveal an underlying mechanism. To provide context, other assessments of emphysema progression in non-HIV cohorts which have used a quantitative rather than qualitative approach through lung densitometry measurements have found that lower BMI[28], current smoking status[29], and female sex[29] are associated with more rapid emphysema progression. Circulating biomarkers such as soluble intercellular adhesion molecule (ICAM-1)[30], surfactant protein D[29] and soluble receptor for advanced glycation end product (sRAGE)[29] have also been shown to be higher in more rapid emphysema progressors.

While our study provides a first step towards the identification of high-risk HIV patients, further work is necessary to implement a useful prognostic tool. First, we report an algorithm with excellent performance characteristics, but this demands that additional validation cohorts be investigated to ensure that the results are generalizable. Our cohort was predominantly male, so further validation will necessitate investigating female HIV populations. Comparisons must also be performed in appropriate non-HIV cohorts, which could address whether emphysema behaves differently in HIV and non-HIV populations. This might also help answer the question as to whether emphysema progresses at a faster rate in HIV. Furthermore, while we have previously reported that emphysema severity is associated with worse SGRQ scores in HIV[4], we do not yet know the clinical significance of emphysema progression. Validating a minimally clinically important difference in qualitative emphysema score would be vital. Because this cohort was made up of HIV patients all currently treated with ART, these findings cannot be extrapolated to untreated HIV patients. Finally, information on exposure to illicit inhaled substances such as marijuana was not collected in this cohort, but could have an effect on lung function and emphysema.

For a disease whose natural history in an aging HIV population is still being defined, our study provides the first longitudinal assessment of emphysema. While many questions including which pathogenic processes drive emphysema progression and whether emphysema progression is accelerated in HIV still require answers, we propose a clinically implementable prediction model to help clinicians identify patients who may experience more rapid worsening of emphysema. Intensive smoking cessation interventions and inhaler therapy may be warranted in these individuals to prevent further progression.

## Supporting Information

S1 Table Other Baseline Variables Not Meeting Statistical Significance In A Univariate Analysis Between Emphysema Progressors and Non-Progressors (DOCX)

S2 Table Antiretroviral Use by Emphysema Progression Group (DOCX)
