# Supplementary material for: Emphysema Distribution and Diffusion Capacity Predict Emphysema Progression in Human Immunodeficiency Virus Infection
Source: PLoS One. 2016 Nov 30;11(11):e0167247. doi: 10.1371/journal.pone.0167247 (PMC5130231; doi:10.1371/journal.pone.0167247)
Supplement: S1 Table — (DOCX) [file pone.0167247.s001.docx]

**S1 Table: Other Baseline Variables Not Meeting Statistical Significance In A Univariate Analysis Between Emphysema Progressors and Non-Progressors^#^**

| **Variable** | **Progressors**  **(n=60)** | **Non-Progressors**  **(n=285)** | **p-value*** |
| --- | --- | --- | --- |
| Total Protein (g/dL) | 7.50 (7.03, 7.95) | 7.60 (7.28, 8.00) | 0.206 |
| CD4:CD8 Ratio | 0.67 (0.56, 0.95) | 0.73 (0.53, 1.03) | 0.268 |
| FVC (L) | 4.61 (4.00, 5.04) | 4.36 (3.79, 5.03) | 0.271 |
| HDL (mg/dL) | 41 (33, 50) | 41 (36, 50) | 0.316 |
| AST (U/L) | 27 (21, 34) | 24 (20, 33) | 0.331 |
| HIV Viral Load <40 copies/mL | 43 (72%) | 224 (79%) | 0.347 |
| History of Myocardial Infarction | 0 (0%) | 9 (3.2%) | 0.369 |
| History of Hepatitis C | 11 (18%) | 45 (16%) | 0.381 |
| Triglyceride (mg/dL) | 169 (126, 254) | 164 (116, 242) | 0.387 |
| Baseline FEV1 % Predicted | 105.4 (93.5, 117.3) | 106.2 (97.3, 116.5) | 0.411 |
| Baseline FVC % Predicted | 111.0 (95.3, 122.6) | 109.0 (98.6, 116.0) | 0.481 |
| LDL (mg/dL) | 117 (97, 145) | 119 (102, 143) | 0.509 |
| Age (years) | 48.5 (45.0, 52.3) | 49.0 (45.0, 54.0) | 0.551 |
| Total Exposure to NRTIs (Months) | 102 (56, 163) | 119 (64, 158) | 0.612 |
| ALT (U/L) | 32 (20, 48) | 29 (21, 47) | 0.648 |
| Total Bilirubin (mg/dL) | 0.78 (0.64, 2.56) | 0.82 (0.65, 1.63) | 0.649 |
| Current CD4 Cell Count (cells/mm^3^) | 574 (209, 646) | 577 (436, 734) | 0.652 |
| FEV1 (L) | 3.49 (3.01, 4.04) | 3.54 (3.02, 4.02) | 0.678 |
| Total Exposure to NNRTIs (Months) | 24 (0.5, 58) | 22 (1, 67) | 0.735 |
| C-reactive Protein (mg/L) | 0.14 (0.10, 0.29) | 0.12 (0.10, 0.25) | 0.738 |
| GFR (Cockcroft) (mL/min) | 94.9 (77.3, 105.9) | 93.0 (78.8, 109.1) | 0.818 |
| Total Exposure to Protease Inhibitors (Months) | 56 (26, 82) | 52 (19, 91) | 0.883 |
| Nadir CD4 Cell Count (cells/mm^3^) | 177 (82, 289) | 190 (65, 290) | 0.895 |
| Total Cholesterol (mg/dL) | 198 (165, 226) | 196 (172, 222) | 0.981 |

^#^Values are recorded as either median (interquartile range) or n (%).

*P-values obtained either through Wilcoxon rank sum testing for continuous variables or Fisher’s exact test for categorical variables.
