# Supplementary material for: Emphysema Distribution and Diffusion Capacity Predict Emphysema Progression in Human Immunodeficiency Virus Infection
Source: PLoS One. 2016 Nov 30;11(11):e0167247. doi: 10.1371/journal.pone.0167247 (PMC5130231; doi:10.1371/journal.pone.0167247)
Supplement: S2 Table — (DOCX) [file pone.0167247.s002.docx]

**S2 Table. Antiretroviral Use by Emphysema Progression Group**

| **Drug Name** | **Drug Abbreviation** | **Emphysema Progressors**  **(n=60)** | **Emphysema**  **Non-Progressors**  **(n=285)** | **p-value*** |
| --- | --- | --- | --- | --- |
| Tenofovir | TFV | 22 (37%) | 127 (45%) | 0.394 |
| Emtricitabine | FTC | 30 (50%) | 127 (45%) | 0.448 |
| Ritonavir | RTV | 22 (37%) | 127 (45%) | 0.394 |
| Lamivudine | 3TC | 24 (40%) | 125 (44%) | 0.781 |
| Atazanavir | ATV | 21 (35%) | 84 (29%) | 0.430 |
| Abacavir | ABC | 14 (23%) | 66 (23%) | 0.999 |
| Efavirenz | EFV | 15 (25%) | 62 (22%) | 0.647 |
| Nevirapine | NVP | 9 (15%) | 41 (14%) | 0.999 |
| Raltegravir | RAL | 4 (7%) | 42 (15%) | 0.158 |
| Darunavir | DRV | 4 (7%) | 42 (15%) | 0.158 |
| Lopinavir | LPV | 4 (7%) | 27 (9%) | 0.690 |
| Zidovudine | AZT | 3 (5%) | 19 (7%) | 0.879 |
| Amprenavir | APV | 5 (8%) | 14 (5%) | 0.433 |
| Etravirine | ETR | 0 (0%) | 10 (4%) | 0.303 |
| Didanosine | DDI | 4 (7%) | 6 (2%) | 0.127 |
| Saquinavir | SQV | 1 (2%) | 5 (2%) | 0.999 |
| Maraviroc | MVC | 1 (2%) | 7 (2%) | 0.999 |
| Tiprinavir | TPV | 1 (2%) | 7 (2%) | 0.999 |
| Enfuvirtide | T20 | 0 (0%) | 4 (1%) | 0.806 |
| Stavudine | D4T | 1 (2%) | 1 (0.4%) | 0.768 |
| Fosamprenavir | FPV | 0 (0%) | 2 (0.7%) | 0.999 |

*P-values obtained by Fisher’s exact test.
